# Development of a Novel *Ex-vivo* 3D Model to Screen Amoebicidal Activity on Infected Tissue

**DOI:** 10.1038/s41598-019-44899-5

**Published:** 2019-06-10

**Authors:** Nancy Elena Guzmán-Delgado, Irma Edith Carranza-Torres, Sara García-Davis, Gildardo Rivera, Javier Morán-Martínez, Nadia Denys Betancourt-Martínez, G. M. M. Groothuis, I. A. M. de Graaf, Pilar Carranza-Rosales

**Affiliations:** 1 División de Investigación en Salud, UMAE, Hospital de Cardiología #34, Instituto Mexicano del Seguro Social, Monterrey, Nuevo León Mexico; 2 Centro de Investigación Biomédica del Noreste, Instituto Mexicano del Seguro Social, Monterrey, Nuevo León Mexico; 3 Facultad de Ciencias Biológicas, Universidad Autónoma de Nuevo León, San Nicolás de los Garza, Nuevo León Mexico; 4 Laboratorio de Biotecnología Farmacéutica, Centro de Biotecnología Genómica, Instituto Politécnico Nacional, Reynosa, 88710 Tamaulipas Mexico; 5 Centro de Investigación Biomédica, Facultad de Medicina, Universidad Autónoma de Coahuila, Torreón, Coahuila Mexico; 6 Groningen Research Institute of Pharmacy, University of Groningen, Groningen, The Netherlands

**Keywords:** Cell biology, Gastrointestinal models

## Abstract

Amoebiasis is a parasitic disease that causes thousands of deaths every year, its adverse effects and resistance to conventional treatments have led to the search of new treatment options, as well as the development of novel screening methods. In this work, we implemented a 3D model of intestine and liver slices from hamsters that were infected *ex vivo* with virulent *E*. *histolytica* trophozoites. Results show preserved histology in both uninfected tissues as well as ulcerations, destruction of the epithelial cells, and inflammatory reaction in intestine slices and formation of micro abscesses, and the presence of amoebae in the sinusoidal spaces and in the interior of central veins in liver slices. The three chemically synthetized compounds T-001, T-011, and T-016, which act as amoebicides *in vitro*, were active in both infected tissues, as they decreased the number of trophozoites, and provoked death by disintegration of the amoeba, similar to metronidazole. However, compound T-011 induced signs of cytotoxicity to liver slices. Our results suggest that *ex vivo* cultures of precision-cut intestinal and liver slices represent a reliable 3D approach to evaluate novel amoebicidal compounds, and to simultaneously detect their toxicity, while reducing the number of experimental animals commonly required by other model systems.

## Introduction

The intestinal parasite *Entamoeba histolytica* (*E*. *histolytica*) is the causative agent of amoebiasis. This disease is the fourth cause of death produced by protozoan parasites^1^. According to the World Health Organization, *E*. *histolytica* infects approximately 500 million people worldwide. However, because of the intra hospital variability in incubation period and disease presentation, only 10–20% of *E*. *histolytica* infections lead to 50 million reported cases of invasive disease and mortality in 100,000 persons each year^2,3^. In some cases, trophozoites destroy the intestinal mucosa and spread to other organs through the bloodstream producing extra-intestinal infections, the most common of which is amoebic liver abscess^4,5^.

For the treatment of intestinal and extra-intestinal amoebiasis, 5-nitroimidazole derivatives such as metronidazole, remain as the first therapeutic option^6^. However, this class of drugs produces undesirable side effects such as nausea, vomiting, diarrhea, hypersensitivity, and may even induce carcinogenesis^7,8^. Since the introduction of metronidazole for the treatment of amoebiasis, few other treatments have been developed. Nevertheless, this disease is still responsible for a high morbidity and mortality rates worldwide, mainly due to the increase in resistance to these and other anti-amoebic drugs^8–10^.

On the other hand, in recent years the interest in designing and synthetizing new antiparasitic molecules has been renewed, facilitated by the application of *in silico* docking; by using such techniques several research groups have investigated newly synthetized molecules as potential antiprotozoal compounds, reporting active molecules against *E*. *histolytica* and other protozoa^11,12^. An example of these bioactive compounds are T-001, T-011, and T-016 (Table 1), which are quinoxaline-7-carboxylate 1,4-di-N-oxide derivatives^11^, and were used in the present work to validate the model reported here. Although the aforementioned compounds possess *in vitro* activity against *E*. *histolytica*, tissue-acting drugs are required to treat invasive amoebiasis, which usually are evaluated in either *in vitro* and/or *in vivo* models. Although *in vivo* models have contributed significantly to the study of amoebiasis, there is no animal model that mimics the whole cycle of the human disease^13^. For most *in vitro* studies, axenic cultures of *E*. *histolytica* are used^14,15^. However, it has been reported that long-term axenic cultivation has a profound effect on *E*. *histolytica* virulence and several virulence markers, and culture conditions induce a strong selective pressure on *E*. *histolytica*, since amoebic virulence can vary from zero to its highest grade, or vice versa^16^. Also, *in vitro* cultures lack complex interactions between stroma components and different cell types that contribute to amoeba pathogenesis in animals. Regarding the latter, *ex vivo* cultures of precision-cut intestinal and hepatic slices represent alternative 3D models that contain all of the cell types belonging to the tissue from which they were obtained, and are therefore considered as mini-organs. Slices have many advantages over *in vitro* and *in vivo* systems, i.e. they retain their metabolic and transport capacity among others^17^. In previous papers, our group reported that hamster liver slices infected with *E*. *histolytica* represent an *ex vivo* 3D model to study hepatic amoebiasis based on morphological and molecular characterization of the disease *ex vivo*^18,19^. In these studies, we observed that morphologic characteristics in amoebae were conserved at the evaluated experimental times, as were eritophagocytic activity, immune response reaction, and virulence factors induction. Furthermore, Bansal *et al*.^20^ reported human colon explants to analyze amoebic virulence factors and reproduce the intestinal invasion by *E*. *histolytica* as well as the tissue non-response to *E*. *dispar*, a non-pathogenic species. Similarly, Girard-Misguich *et al*.^21^ utilized porcine colonic explants, derived from miniature histocompatible SLA^d/d^ piglets, to co-culture with virulent (HM1:IMSS) and avirulent (Rahman) strains of *E*. *histolytica*. They were able to observe the tissue invasion by the trophozoites and the typical innate immune response, similarly to the report on human colon explants. Likewise, Ximenez *et al*.^22^ evaluated genes related to the pathogenicity and *E*. *histolytica* survival, and genes linked to the immune response in precision cut liver slices from human tissue. The afore mentioned studies and the present work differ between them in the origin of the tissue employed (hamster, pig or human), explant preparation methodology (manual or semiautomatic), culture media, amoeba strains (virulent/avirulent), trophozoite inocula, and evaluated genes (host or parasite genes).

**Table 1:** Amoebicidal activity and cytotoxicity of the synthetic compounds.

Compound*	Chemical Structure	IC_50_ *vs E*. *histolytica*	IC_50_ *vs* Vero Cells	SI
T-001		1.41	>100	>70.92
T-011		0.35	5.86	16.74
T-016		1.47	>100	>68.02
Mtz		4.50	>100	>22.22

^*^Duque-Montaño *et al*., 2013.

IC_50_ in µM.

Mtz: Metronidazole.

In spite of the technical differences, tissue explants have been successfully used to reproduce the infections with *E*. *histolytica*^20–22^ and have the advantage to apply one of the principles of the 3 R’s philosophy, reducing the number of animals for experimentation.

With the acquired experience using liver slices as 3D cultures, in the present work we implemented the *ex vivo* culture of intestinal slices as an infection model of *E*. *histolytica*. Moreover infected precision-cut hamster liver and intestinal slices were used to analyze anti-amoebic activity, and at the same time, the possible toxicity of the amoebicidal molecules T-001, T-011, and T-016 in the infected tissue, in comparison to metronidazole as a reference compound.

This experimental approach allowed us to investigate effectivity of the tested compounds against *E*. *histolytica* in tissue, and demonstrates the feasibility of the model for the research of new antiprotozoal drugs.

## Results

### Amoebicidal and cytotoxic effect of T-001, T-011, and T-016

All three compounds, T-001, T-011, and T-016 were effective against *E*. *histolytica*, with IC_50_ values < 2 µM. Compound T-011 was cytotoxic *in vitro* against Vero cell cultures (IC_50_ = 5.86 µM), whereas T-001 and T-016 were not cytotoxic (IC_50_ > 100 µM), resulting in selectivity index (SI) > 65; these two compounds have anti-amoebic activity and SI higher than the reference drug metronidazole (IC_50_ = 4.5 and a SI > 22.22). These results are summarized in Table 1.

### Obtaining precision-cut hamster intestinal slices

**Figure 1:**
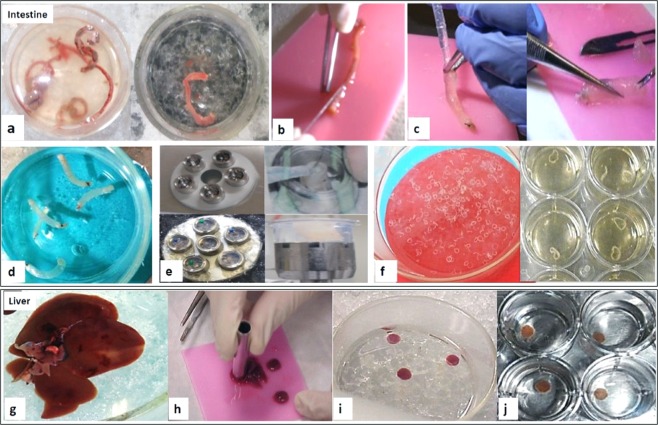
Preparation of hamster tissue slices. To obtain intestinal slices (upper panel), all the steps were performed under aseptic conditions, in no more than 1 h. **(a)** After dissection, the intestinal content was washed with cold KB buffer and the colon was divided in 3 cm segments; clean fragments were placed into ice-cold KB buffer. **(b)** The adhering fatty tissue was carefully removed. **(c)** One side of the intestinal fragments were tied with surgical thread and filled with 3% low melting point agarose at 37 °C. **(d)** Agarose infiltrated fragments filled with agarose were transferred into ice-cold KB buffer to permit the agarose solidification. **(e)** The intestinal segments were collocated in the tissue embedding unit of the Krumdieck tissue slicer and a pin was placed in the solid agarose to help the filling with 2% low melting point agarose at 37 °C of the mold in order to form cylinders containing the colon segments. **(f)** Colon tissue slices (300–400 µm thickness) were prepared using the Krumdieck tissue slicer and were collected in ice-cold KB buffer and incubated in culture medium at 37 °C. The process to obtain liver slices (lower panel) is simpler than intestinal slices; the process was also performed under sterile conditions, and in the shortest possible time. **(g)** After the liver was removed, it was placed into ice-cold KB buffer and the hepatic lobes were separated with a scalpel. **(h)** The hepatic lobes were cored into 5 mm diameter cylinders. **(i)** The cores were then placed in oxygenated KB buffer (4 °C, 95:5 O_2_: CO_2_). **(j)** Liver tissue slices with 250–300 µm thickness were prepared using the Krumdieck tissue slicer and were gently placed and incubated onto 24-well microplates with culture medium at 37 °C.

**Figure 2:**
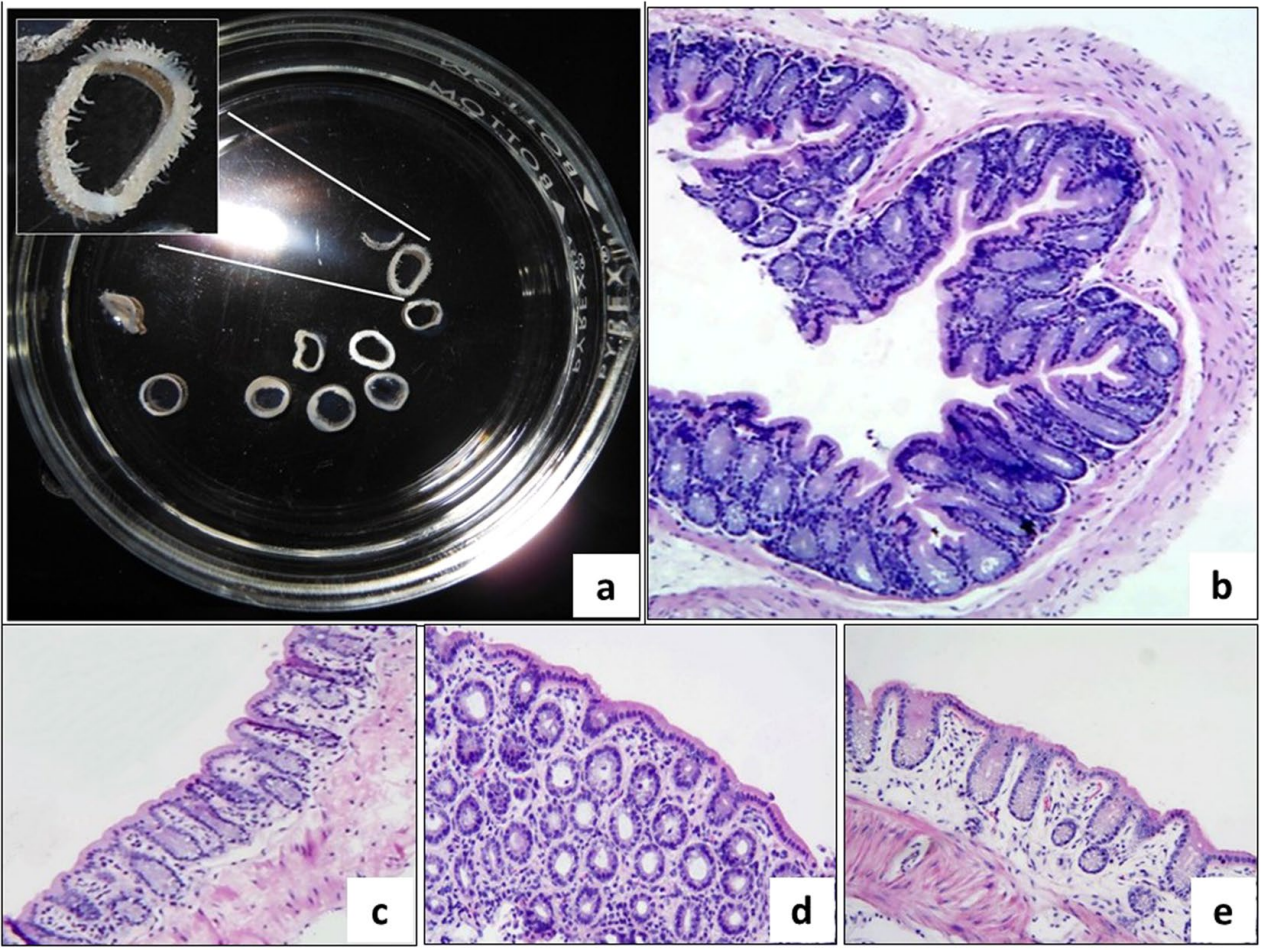
Macroscopic and microscopic view of intestinal slices. (**a)** Panoramic view of freshly obtained intestinal slices showing the typical aspect of intestine fragments cut transversally and, in the insert, a close view of the brush border microvilli; **(b–e)** Microphotographs shows colonic mucosa with well-preserved histologic architecture during different incubation time, 0, 3, 6 and 9 h, respectively. H&E stain (Total magnification: 100X).

The preparation and culture of complete tissue slices from hamster intestine and liver was successfully established using the Krumdieck tissue slicer (Fig. 1). The slices prepared in this way maintained histological and morphologic characteristics of the normal intestine, preserved intestinal microvilli (Fig. 2a), and remained viable during the incubation period (Fig. 2b–e).

### Infection of precision-cut hamster intestinal slices with *E*. *histolytica*

**Figure 3:**
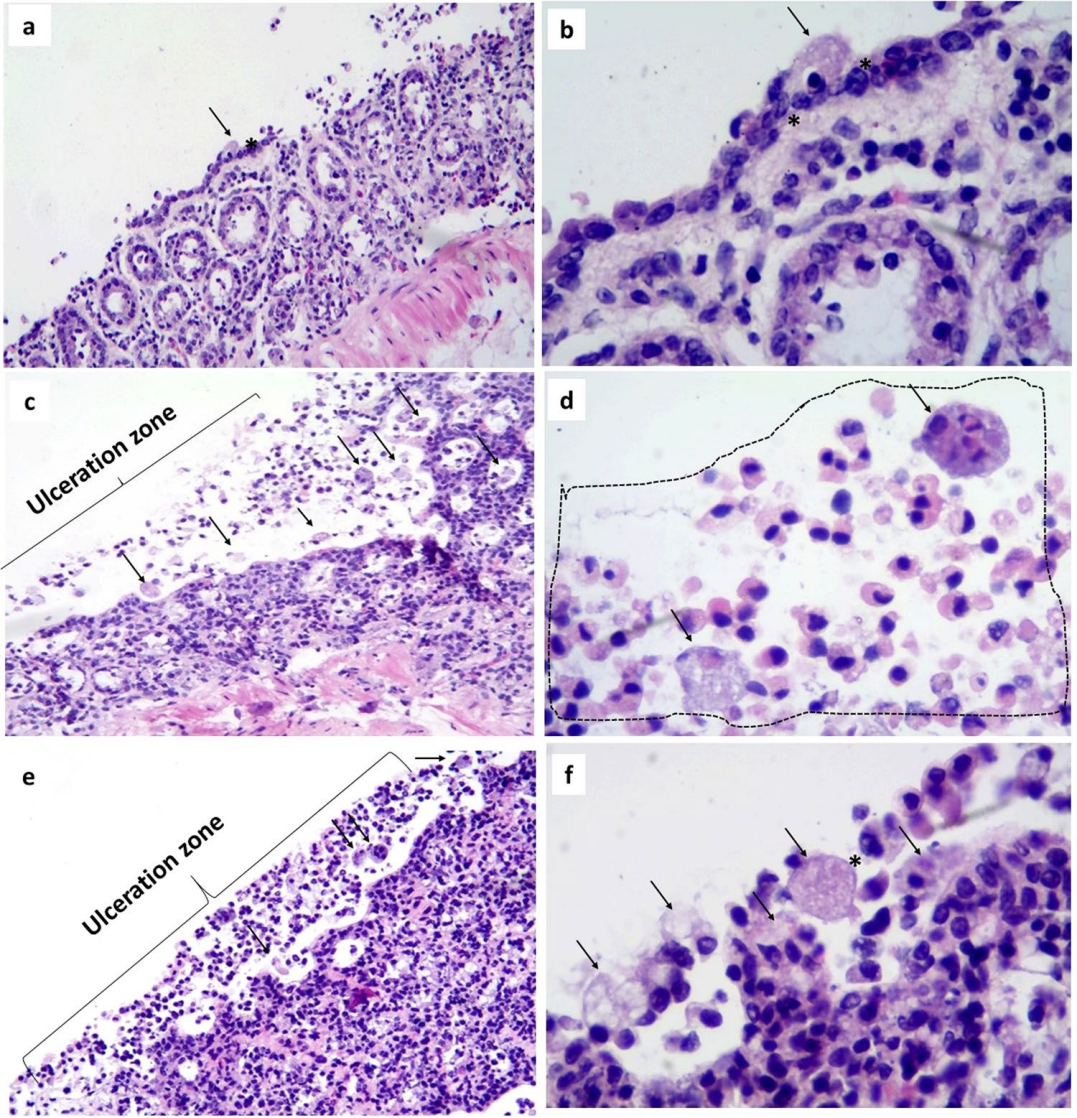
Experimental infection of intestinal slices cultured *ex vivo*. These microphotographs show the infection of the colonic mucosa with *Entamoeba histolytica* trophozoites in slices that were cultured for 3 h **(a,b)**, 6 h **(c,d)**, and 9 h **(e,f)**. The most remarkable morphologic change is the ulceration of the mucosa as a consequence of the infection process; morphologically well conserved amoebae (arrows) are in close contact with epithelial cells (*) and are able to induce a severe acute inflammatory process composed mainly by neutrophils and eosinophils (dotted area in Fig. d). Figures b, d, and f correspond to microscopic magnifications of figures a, c, and e, respectively. H&E stain (Total magnification: 100X, 400X).

Experimental infection of the intestinal slices was also achieved successfully. Figure 3 shows ulcerations in the mucosa of the colon due to infection with the trophozoites of *E*. *histolytica*. Amoebae were frequently observed in close contact with the epithelial cells and subsequently penetrated the mucosa in a massive way, inducing a marked acute inflammatory response characterized by the presence of polymorphonuclear cells and eosinophils. All of these morphological events related to the experimental infection were observed indistinctly at 3 h (Fig. 3a,b), 6 h (Fig. 3c,d) and 9 h (Fig. 3e,f). It is important to mention that under our experimental conditions, amoebae remained viable and healthy during the infection assays, as judged by their invasive ability, pseudopodia formation and change of shape inside the stromal tissue, as well as elevated erythrophagic activity, and the induction of characteristic tissue lesions observed in intestinal amoebiasis.

### Amoebicide effect of T-001, T-011, and T-016

**Figure 4:**
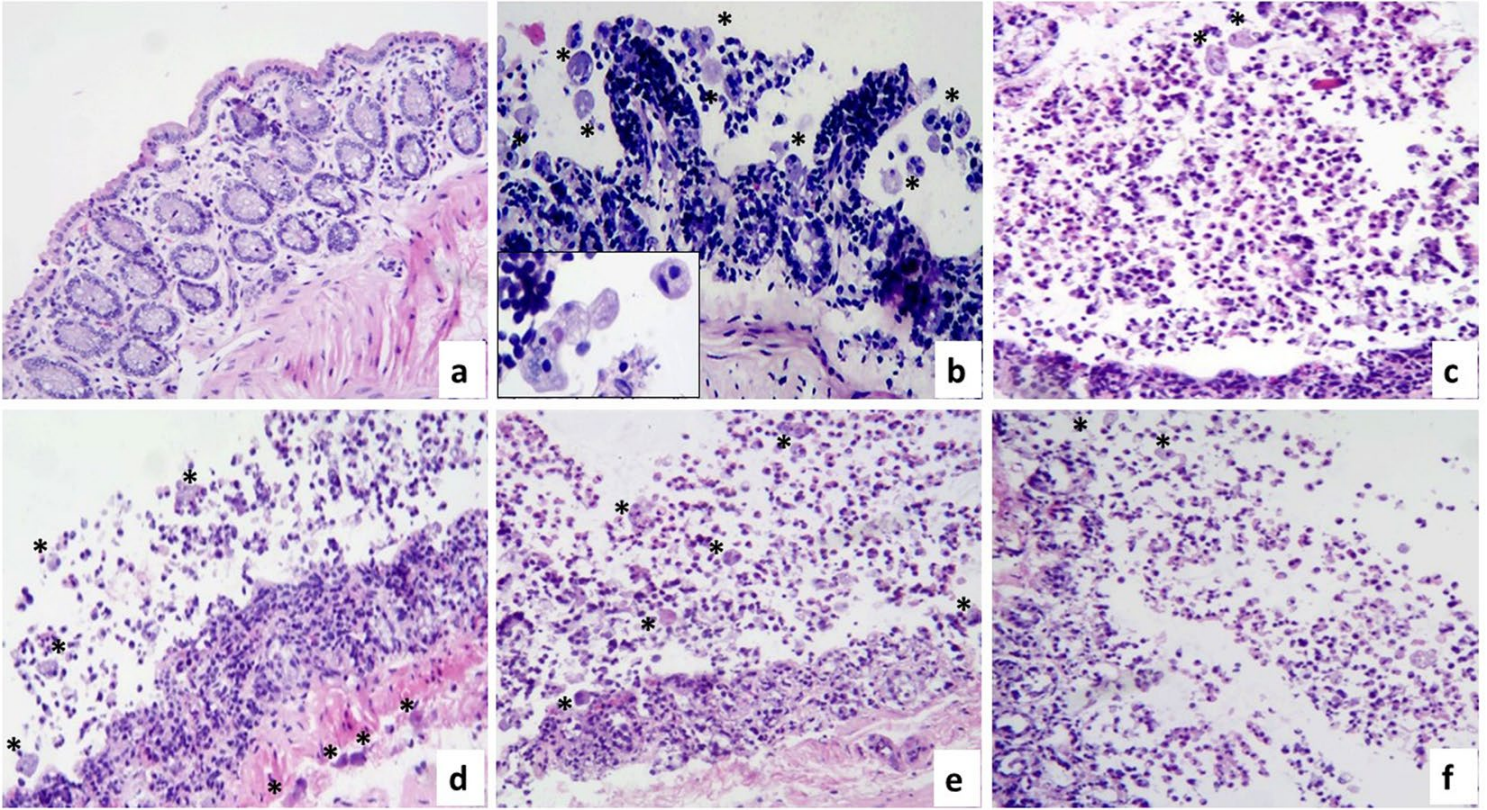
Amoebicide effect of T-001, T-011 and T-016 on hamster intestinal slices. Microphotographs show intestinal tissue cultured for 9 h. **(a)** Negative control, intestinal mucosa without morphological alterations; **(b)** infection control showing the presence of conserved amoebae (*) at the intraluminal level (insert), as well as severe acute inflammation, ulceration of the intestinal mucosa and intracryptic abscesses; **(c)** in the pharmacological control (metronidazole) dirty background is observed due to the presence of cellular debris coming from inflammatory cells, fragments are also identified and alteration of amoebic structures (arrows) in comparison with trophozoites showing erythrophagy (*). In the slices incubated in the presence of **(d)** T-001, **(e)** T-011, and **(f)** T-016 damage to amoebae (arrows) was also observed, which is characterized by amoeba fragmentation, although slightly less than pharmacological control. Compound T-011 at the used concentration showed greater amoebicidal tendency than T-001 and T-016. In non-infected intestinal slices incubated only with the synthetic compounds, morphological changes suggestive of toxicity were not observed (data not shown). H&E stain (Total magnification: 100X and 400X).

**Figure 5:**
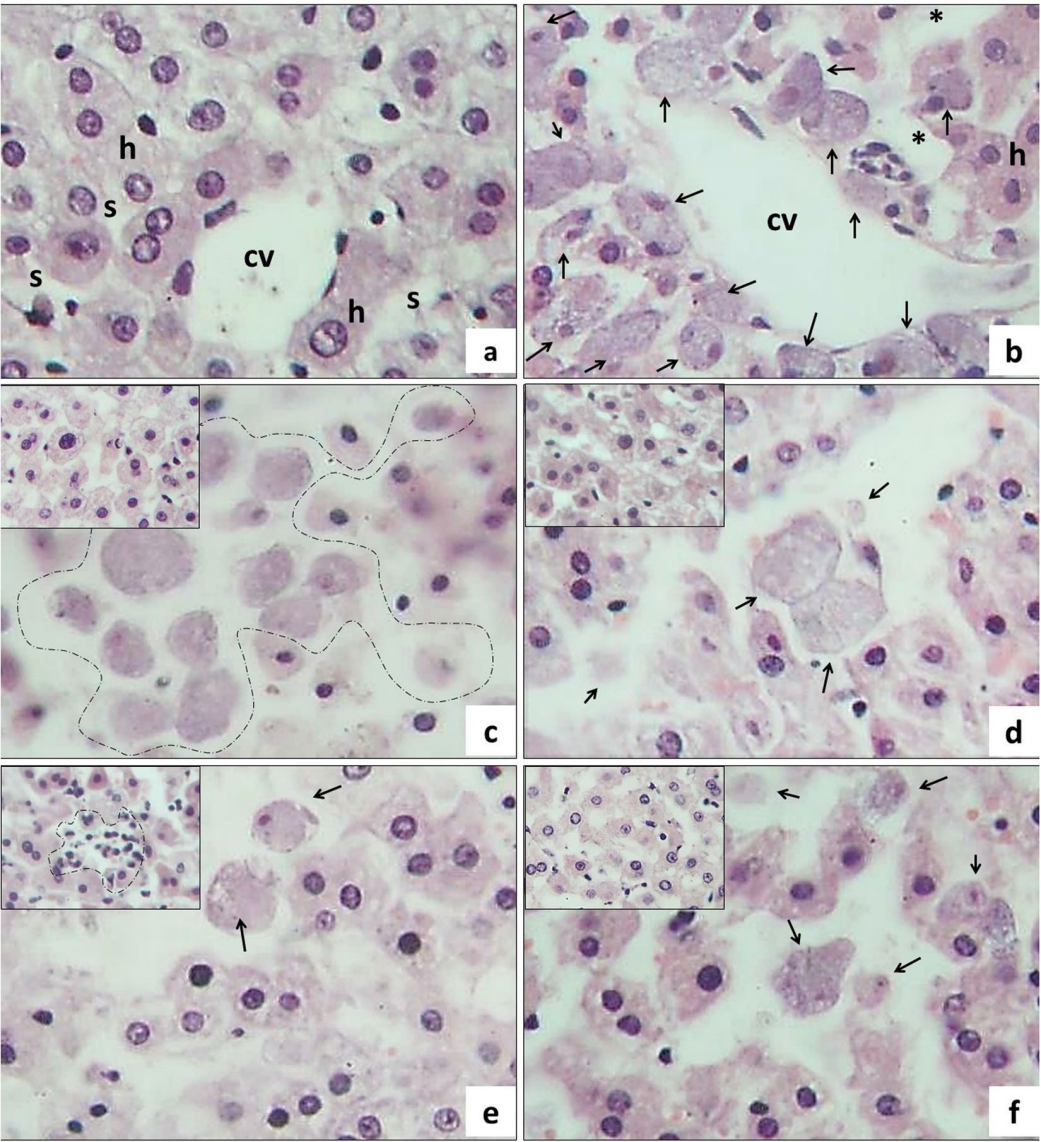
Amoebicide effect of T-001, T-011, and T-016 on *ex vivo* cultures of hamster hepatic slices. Non-infected (control) liver slices **(a)** shows the typical histological structure of hepatic tissue: hepatocytes (h), sinusoidal space (s) and central vein (cv). **(b)** Liver slices infected with trophozoites shows numerous amoebae (arrows) and a pronounced dilatation of the sinusoidal spaces (*). The amoebicide effect of metronidazole **(c)** is evident by a diminution in the number of amoebae per microscopic field; most of them were dead and in process of disintegration (dotted line). **(d)** T-001, **(e)** T-011, and **(f)** T-016, also induced a decrease in the number of amoebae and provoked similar morphological damage to metronidazole (arrows), and in addition, induced inflammatory reactions (dotted area in the insert of Fig. e). Non-infected slices were also incubated in the presence of the synthetic compounds (top left figure inserts in d–f), while T-001 and T-016 induced regenerative changes and cytoplasmic vacuolization, T-011 induced focal hepatocellular necrosis and acute inflammation (dotted line in the insert). H&E stain (Total magnification: 400X).

As we expected, the synthetic compounds showed *ex vivo* amoebicide effect in both, intestine (Fig. 4), and liver (Fig. 5) slices infected with the virulent trophozoites of *E*. *histolytica*, and their amoebicidal effect was comparable to metronidazole. In both tissues, the compounds induced a marked decrease in the number of trophozoites, and fragmentation/disintegration of the amoebae.

With regard to the anti-amoebic effect in intestinal slices, representative images of control and infected slices cultured during 9 h in presence or absence of compounds are shown in Fig. 4. In the slices cultured only with culture medium (negative control) the histologic structure is well preserved, (Fig. 4a); meanwhile in the infected slices, numerous viable amoebae at the intraluminal level, severe acute inflammation, ulceration of the intestinal mucosa, and intracryptic abscesses were frequently observed (Fig. 4b). As expected, the pharmacological control (metronidazole) induced death of the amoebae and therefore decreased their number (Fig. 4c), with this treatment a “dirty background” due to cell debris derived from inflammatory cells was usually present. In the intestinal slices infected and incubated with the synthetic compounds (Fig. 4d–f), a decrease in number of amoebae per field was also observed, in a similar way to metronidazole. In the infected slices incubated in the presence of the synthetic compounds, amoebae in disintegration process were observed, but in less extension than metronidazole. Compared to compounds T-001 and T-016, T-011 showed greater amoebicidal effect on the infected tissue.

**Figure 6:**
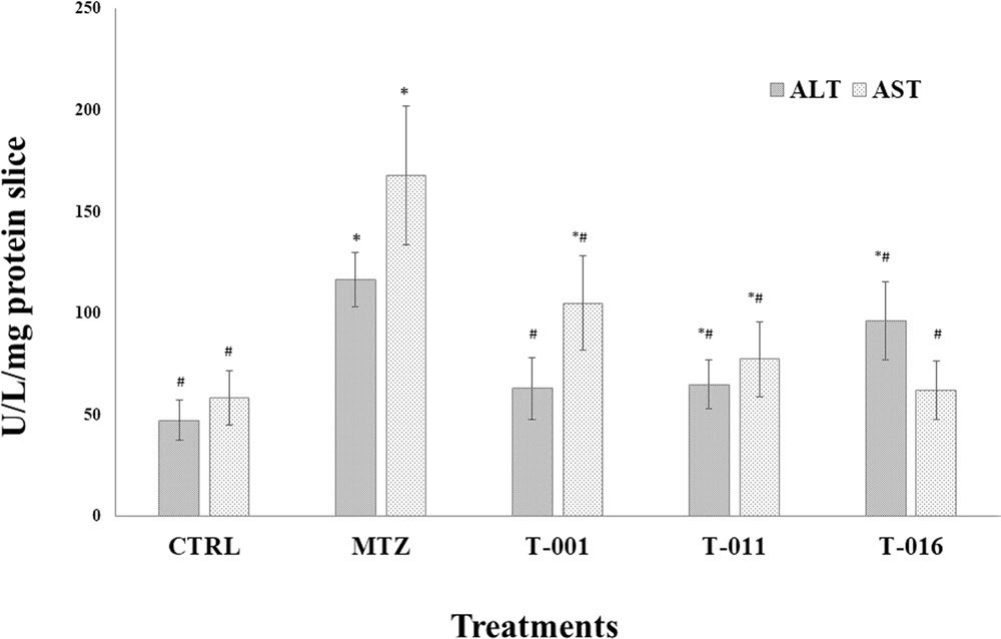
Tissue toxicity of T-001, T-011, and T-016 on precision-cut hamster liver slices. Hepatic ALT and AST levels were measured after 24 h incubation of tissue liver slices in the presence of the three synthetic compounds, MTZ and control negative (culture medium). Each line represents the group mean ± S.D. (n = 3). Asterisk (*) indicates significant difference (p < 0.05) compared with control (−) and, hash (#) indicates significant difference (p < 0.05) compared with positive control (+) MTZ in each sample.

In liver slices infected with *E*. *histolytica* trophozoites and treated with the synthetic compounds T-001, T-011 and T-016 (Fig. 5d–f, respectively), a marked decrease in number of amoebae per microscopic field and fragmented amoebae, as well as acute inflammatory response cells were frequently seen. The anti-amoebic effect was similar to metronidazole (Fig. 5c). When non-infected slices were incubated in presence of the synthetic compounds, T-001, and T-016 induced regenerative/adaptive changes and microsteatosis accompanied by increased AST and ALT levels, respectively; T-011 provoked focal hepatocellular necrosis and acute inflammation (see top left inserts in Fig. 5d–f), which was reflected in increased levels of both ALT and AST. Nevertheless, compared to metronidazole, the three compounds were significantly less hepatotoxic (Fig. 6).

## Discussion

*E*. *histolytica* is an enteric protozoan that causes human amoebiasis, which is considered the fourth cause of parasitic diseases^23,24^. Metronidazole (a nitroimidazole derivative) is often the selected drug to treat amoebiasis, however, this drug is mutagenic, carcinogenic and genotoxic^25^ and has many other undesirable side effects, amongst others neurotoxicity^26,27^. Likewise, the appearance of strains of *E*. *histolytica* resistant to conventional drugs has been reported^28^.

Given the aforementioned, the search for new anti-amoebic agents continues to be a global trend, for which different approaches have been taken, from the synthesis of new compounds^29,30^ to the systematic study of natural products^14,31^. Anti-amoebic activity is traditionally tested *in vitro* using axenic strains of *E*. *histolytica*^32–34^; in addition, and taking into account the necessary breakdown of intercellular structures for infection, cell lines such as Caco-2, T84 and MDCK have been used for interaction studies during amoebiasis^35^. *In silico* analysis are also used^36–38^.

Although the use of *in vitro* and *in silico* assays allow the screening of a large number of molecules in record time, the scope and limits of the results obtained have been questioned since these models reflect the *in vivo* situation only partially, and do not allow studies of drug-parasite-host interactions^39^, which is of great importance for the study of adherent parasites such as *E*. *histolytica*. Animal models also have been used for the study of amoebiasis, however they have the disadvantage that is not possible to induce both intestinal and hepatic lesions in the same animal host^13^. Since *E*. *histolytica* only infects humans and non-human primates, results obtained in *in vivo* studies with laboratory animals cannot always be translated directly to humans. The practical application of using *in vivo* tests for the investigation of new compounds or molecules with anti-amoebic activity is limited by both, the large number of animals that would be needed, and by the large amount of compounds required.

Because of the above mentioned we implemented the use of precision-cut intestinal and hepatic tissue slices as an alternative model to study anti-amoebic activity of compounds with potential effect against *E*. *histolytica* using the organs from the same animal. Precision-cut intestinal and hepatic tissue slices are considered an *ex vivo* 3D model with intermediate characteristics between *in vitro* and *in vivo* systems^40^, they are also considered as mini-organs with metabolic capacity to transport and metabolize chemical drugs^17,41,42^.

In the present work, we describe in detail the implementation of the *ex vivo* infection model of hamster intestine slices with *E*. *histolytica* trophozoites, since previously we reported the usefulness of hamster liver slices as an infection model with the same parasite. Results using liver slices infected with amoebae in the currently study are identical to those obtained in our previous work^18,19^. We also validated intestinal and hepatic slices as experimental infection models using compounds with known anti-amoebic activity. In the current study, intestinal slices were cultured up to 9 h in the presence of the amoebae (12 h of incubation in total). Other investigators have reported maintenance of intestinal slice viability during similar culture periods. For example, de Kanter *et al*.^43^, and van de Kerkhof *et al*.^44^ reported maintenance of viability and metabolic activity up to 3 h of culturing. Two hours of incubation was used to analyze the interaction of xenobiotic agents with transporting proteins in the intestine^45^. Other authors have shown that small and large intestine slices remain viable for 8 and 24 h respectively when the intracellular ATP content was evaluated, while after 24 h most of the phase I and II enzymes implicated in metabolic transformation show significant changes depending on the origin of the tissue^46^. The incubation time over which tissue slices remain viable depends on the tissue, where 96 and 24 h are suitable for liver slices and intestinal slices respectively^47^. In general terms, we were able to observe the typical lesions of the amoebic disease during the *ex vivo* infection of both PCLS and PCIS. Incubation during longer times with the virulent strain of *E. histolytica* caused massive destruction of both tissues due to the amoebae infection.

To ensure that the amoebicides T-001, T-011 and T-016 are selectively toxic towards the amoebae, we analyzed their cytotoxic effect *in vitro* on Vero cells, a well-characterized kidney derived cell line that is not tumorigenic below particular passage numbers^48^ and therefore, it is frequently used as a normal cell line to determinate toxicity during preliminary screens of several compounds with biological activity^15,49^. Since Vero cells are useful to select those compounds that can be further tested or characterized in more complex systems, we found that the concentrations used in our tests were not harmful *in vitro* (IC_50_ > 100 µM). One of the compounds, T-011, showed *in vitro* cytotoxicity at a concentration of 5.86 µM, however to decrease the cytotoxic effect, the concentration of this compound may be reduced since the IC_50_ value vs *E*. *histolytica* is 0.35 µM.

One of the main challenges in the *ex vivo* intestinal tissue culture is the maintenance of viability^50^. However, in the present study the intestine and liver slices were cultured for 12 and 24 h respectively for the infection assays, maintaining metabolic viability as evaluated by the Alamar blue assay (data no shown) and preserving the morphological structure without alterations. Our results are similar to those reported by van de Kerkhof *et al*.^51^, and by Bansal *et al*.^20^, who have maintained *ex vivo* tissue cultures during 3 h and 7 h, respectively.

The first pathologic signs that we observed during the experimental infection of the intestinal tissue slices were ulceration of the mucosa and acute inflammation, whilst liver slices showed dilatation of the sinusoidal spaces, and micro abscesses. Numerous viable amoebae were seen in both infected tissues. Even though tissue slices have a lack of bloodstream, the observed inflammatory process could be related to a specific pattern of endogenous chemoattractants and inflammatory chemokines produced by immune and cells present in the intestine tissue when tissues were collected, since it is known that the gastrointestinal mucosa is the largest reservoir of macrophages in the body^20,52^. In the same way, Bansal *et al*.^20^ reported the presence of monocytes and T-lymphocytes within the lamina propria of colonic explants, as well as the recruitment and migration of neutrophils and other resident immune cells to the site of infection which contributed to the immune response in intestine explants. This suggests that intestinal tissue slices remain immunologically actives.

The fact of observing histopathological changes from 3 h of interaction between *E*. *histolytica* with the intestinal and hepatic tissue slices suggests that the infection and invasive process starts before this time. In addition, intracryptic abscesses were observed at 9 h of incubation. These changes are similar to those reported by Houpt *et al*.^53^ in C3H/Hej mice infected with *E*. *histolytica*; they also observed viable amoebae in areas of epithelial ulceration and severe inflammation as reflected by the presence of plasma cells, neutrophils, and mast cells. Likewise, *E*. *histolytica* trophozoites degraded the intestinal epithelium and penetrated the mucosa of human intestine explants after 7 h of incubation^54,55^. In addition, Bansal *et al*.^20^ reported that trophozoites reach the epithelial surface and remove the mucosa, detaching the enterocytes and migrating along the crypts. These results are similar to those obtained in the present work. Indeed, the same microscopic changes were described in both *in vivo* and *ex vivo* models since 1970 by Prathap and Gilman, and by Espinosa-Cantellano and Martínez-Palomo for human amoebic ulcerative colitis^56^. Our results lead us to believe that the experimental model of intestinal and hepatic tissue slices can be a valuable tool to predict the effect of anti-amoebic compounds in the early stages of *E*. *histolytica* infection. Although tissue slices have been well accepted for xenobiotic toxicity and metabolism studies^57,58^, as well as models for *ex vivo* infection with viruses^59,60^, bacteria^39^, and parasites^20,61^, the present work represents the first report of the *in situ* effect of compounds with anti-amoebic properties on the progression of infection by *E*. *histolytica* in organotypic culture of both, hepatic and intestinal tissues derived from the same animal. Therefore, a significant reduction of biological variability into the experimental repetitions due to the biological responses between animals and even within the same animal is possible^62^.

It is currently well known that liver slices represent a mini liver model with all the different cell types in their original microenvironment, having metabolic activity, whereas most cell lines have not^63^, and therefore represent a more suitable *ex vivo* model for measuring liver damage induced by different agents^64^. Since hepatotoxicity is an adverse reaction that may be induced by novel compounds being studied, models capable of predicting hepatic toxicity are necessary. Given that ALT and AST enzymes are considered as sensitive biochemical indicators of cellular injury and metabolic disturbances^65^ we determined both enzyme levels on the hepatic slices treated with the synthetic compounds and metronidazole. Increased levels of ALT and AST enzymes indicate a toxicological effect of the tested compounds^66^, therefore, their increase in tissues treated with T-011 confirm the cytotoxicity found on Vero cells (IC_50_ = 5.86 µM) and their lower selectivity (SI 16.74) compared to tissues treated with T-001 and T-016, which are not toxic to Vero cells and exhibited higher selectivity (SI > 65). Also, T-011 Induced more pathological changes at histologic level. T-001 and T-016 induced less inflammation and necrosis, and raised one of the two hepatic enzymes. Nevertheless, compared to metronidazole, the three compounds were significantly less toxic in tissue cultures at the used concentrations.

Although compounds T-001 and T-016 did not exert cytotoxic effects on Vero cell (IC_50_ > 100), it is well known that *in vitro* tests using cell monolayers are limited models because they are very different from the start, since generally they are dedifferentiated and lack, amongst others of transport systems and metabolic enzymes^67^ as well as of the ability to maintain histotypic and phenotypic characteristics overtime^68^. The latter may explain the discrepancies observed between *in vitro* studies and 3D models, as in the present work where the active compounds showed a modest cytotoxic effect on the intestine and liver slices at much lower concentrations than where cytotoxicity was seen in the cell line. Similarly, van de Kerkhof *et al*.^51^ showed a high metabolic rate in intestinal tissue slices that can be up to 3 times higher than that estimated in hepatocytes in liver tissue slices.

As it has been said, 3D cultures have great potential to fill the gap between cell-based research and animal models to study host-pathogen interactions, as well as contribute to the development of new treatments^69^. The usefulness of organotypic cultures to understand the pathogenesis of different diseases, as well as their potential as preclinical models, has led to subsequent applications of similar models that had already been validated^20,55^. The use of these *ex vivo* models responds to the increasingly clear need to use physiologically more relevant systems and perform better *in vitro-in vivo* extrapolations (IVIVE)^68^ for many different diseases, like amoebiasis.

In conclusion, our study demonstrate that precision-cut hamster intestinal and liver slices infected with *E*. *histolytica* trophozoites represent a 3D model that allows to simultaneously evaluate both anti-amoebic potential of new compounds, and their possible toxicity. Using this model we showed that T-001, T-011, and T-016, novel synthetic compounds reported as amoebicidal are equally effective against *E*. *histolytica* as the golden standard metronidazole currently used for treatment of the disease, without compromising tissue viability to great extent.

## Materials and Methods

### Animals

The study protocol was approved (Approval: R-2012-785-020) by the National Committee for Scientific Research from the Instituto Mexicano del Seguro Socia (IMSS). Two month old male Syrian golden hamsters (*Mesocricetus auratus*) obtained from the animal facility of the Northeast Biomedical Research Center (IMSS), Monterrey, México were used. The animals were kept in polycarbonate cages, with free access to food and water. Animal handling was in accordance with the international principles on ethical management and care, and according to the Official Mexican Norm NOM 062-ZOO-1999: Technical Specifications for the Production, Care and Use of Laboratory Animals, the guidelines from the Institutional Biosecurity Committee (IMSS), and the Manual of Procedures and Recommendations for Animal Research, 2012. All methods were performed in accordance with these approved guidelines.

### *E*. *histolytica* culture

Virulent trophozoites from *E*. *histolytica* strain HM1-IMSS were cultured and maintained axenically in TYI-S-33 (Trypticase-Yeast extract-Iron-Serum) medium supplemented with 10% bovine serum, penicillin-streptomycin, and Diamond® vitamins. The subculture and the bioassays were performed when the amoebae were in the logarithmic phase of growth.

### Amoebicidal compounds

The synthetic compounds T-001, T-011, and T-016 were synthetized and identified according to the methods reported by Duque-Montaño *et al*.^11^.

### Cytotoxicity of the compounds T-001, T-011, and T-016 on Vero cells

As a preliminary step to the analysis of the anti-amoebic activity of the synthetic compounds on the intestinal and hepatic tissue slices, their possible cytotoxicity on Vero (ATTC®CCL-81™) cell cultures was evaluated. This cell line is used worldwide to assess the *in vitro* cytotoxic effect and/or selectivity index of different compounds^70–74^. Additionally Vero cells are endorsed by ISO experts to be suitable to test cytotoxicity of certain compounds and materials of medical devices^75^.The cell line was cultured in Eagle’s Minimum Essential Medium supplemented with 10% fetal bovine serum and 1% (v/v) of a mixture of 100 IU/mL penicillin/50 µg/mL streptomycin. For the cytotoxicity analysis, confluent logarithmic growth phase cultures were used after 10,000 cells/100 μL were seeded in each of the wells of a 96-well plate. The microplate was incubated overnight and then the compounds were added in a concentration range of 0.1 to 100 μM and incubated for an additional 48 h at 37 °C, in a humid atmosphere and 5% CO_2_. The viability of the cells incubated in the presence of T-001, T-011, and T-016 was determined by the WST-1 assay, which is directly proportional to the number of viable cells^76^. The assay was carried out in duplicate.

### Precision-cut hamster intestine slices preparation

The entire process was performed at 4 °C under sterile conditions, in the shortest time of surgical manipulation in order to minimize the autolysis of the intestinal tissue due to ischemia. Briefly: the hamsters received an intraperitoneal injection of sodium pentobarbital (64 mg/ 100 g of weight). Once in deep anesthesia, the colonic portion of the large intestine was carefully removed and divided into fragments of three centimeters long. These fragments were placed into Krebs-Henseleit buffer (KB buffer) at 4 °C, and were washed with the same buffer to remove the intestinal content, and then the superficial adipose tissue was carefully removed. Afterwards, the intestinal fragments were tied using surgical thread at one end, while on the opposite side was filled with low melting point agarose at 37 °C and placed in KB buffer at 4 °C for the agarose solidification. The fragments of intestine filled with agarose were placed in the tissue embedding unit of the Krumdieck tissue slicer in order to prepare regular agarose cylinders. These cylinders were placed in the sample holder of the Krumdieck tissue slicer, and intestinal tissue slices with thickness of 300–400 μm were prepared, always under constant recirculation of KB buffer at 4 °C gassed with carbogen (95% O_2_/5% CO_2_). The slices were collected in KB buffer at 4 °C to remove the external ring of agarose with aid of a spatula. Figure 1 shows general aspects of the process to prepare both intestinal and hepatic slices.

### Culture and *ex vivo* infection of intestinal slices

Once the intestine slices were obtained, those with the best macroscopic appearance were selected. Subsequently, two slices were placed per well of a 24-well microplate, each well containing 1 mL of DMEM/F12 medium supplemented with 10% fetal bovine serum, 25 mM glucose and 10 mg/L gentamicin and subsequently pre-incubated for 1 h at 37 °C in a cell culture incubator to stabilize the tissue. The intestinal slices were infected with 200,000 virulent trophozoites of *E*. *histolytica* suspended in 2 mL of 1:1 mix of TYI-S-33 medium and complete DMEM/F12 medium. As control, non-infected slices were used. The microplate was incubated resting during 2 h at 37 °C to allow the adherence of the amoebae with the tissue. After this time, the slices were transferred under sterile conditions to another 24-well microplate containing 1 mL of the 1:1 mix of TYI-S-33 and DMEM/F12 media and two washes were performed with KB buffer, to eliminate amoebae that were not attached to the intestinal tissue; infected slices were incubated in 2 mL of the same medium at 37 °C and 95% O_2_/5% CO_2_ under agitation at 30 rpm. Samples were taken at 0, 3, 6, and 9 h for subsequent analysis.

### Preparation, culture and infection of hamster liver slices with *E*. *histolytica*

The obtaining and *ex vivo* culture of hamster liver slices, as well as their subsequent experimental infection, was carried out according to the protocols previously described by our research group^18,19^.

### Anti-amoebic activity of T-001, T-011, and T-016 on precision-cut hamster intestinal and liver slices

After the *ex vivo* infection of the intestine and liver slices with *E*. *histolytica* trophozoites, slices from both tissues were pre-incubated separately in microplates with 1 mL of complete culture medium for 3 h and then, 1.41 μg/mL T-001, 0.35 μg/mL T-011, and 1.47 μg/mL T-016 were added in triplicate wells. These concentrations correspond to the IC_50_ of each compound previously reported *in vitro* by Duque-Montaño *et al*.^11^. Metronidazole (IC_50_ = 4.5 μg/mL) was used as the reference amoebicide drug, whereas 0.3% DMSO and culture medium were used as solvent control and negative control, respectively. The infected and control slices, as well as those treated with the synthetic compounds, were incubated at 37 °C with 95% O_2_/5% CO_2_ and agitation at 30 rpm for 3, 6, and 12 h for intestinal tissue, and 3, 6, 12 and 24 h for liver tissue. At the end of each incubation, slices were fixed in 10% neutral formalin for histopathological analysis. Three independent experiments were performed.

### Hepatotoxicity of T-001, T-011, and T-016

Cell-specific biomarkers analyzed included AST (aspartate aminotransferase) and ALT (alanine aminotransferase) for liver parenchyma. ALT and AST are enzymes considered as sensitive biochemical indicators used to evaluate cellular injury, metabolic disturbances, and enzyme inactivation or induction by exogenous chemicals; and their presence in the liver effluent is a reflection of hepatocyte lysis as consequence of chronic inflammation and other sources of liver injury^65,77^. To evaluate the possible tissue toxicity of the synthetic compounds, liver slices were incubated for 24 h in the presence of the same concentrations used in the infection assays. After incubation, hepatic enzymes ALT and AST were measured as described by Behrsing *et al*.^78^. Briefly: liver slices were removed from the microplate wells and gently rinsed with PBS, afterwards, each slice was transferred into 2.0 ml Potter-Elvehjem tissue grinder containing 0.5 ml PBS + 0.5% Triton X-100 on ice to homogenize the slices. The resulting lysates were centrifuged at 9000 g for 5 min to remove particulate matter. The resulting supernatants were analyzed on a Hitachi 911 clinical analyzer. Three independent experiments were performed.

### Histopathological analysis

After each incubation time the slices were washed with PBS at 37 °C and fixed in 10% buffered formalin for 24 h at room temperature. After a dehydration and clarification process, paraffin blocks were prepared for each treatment and sections of 5 μm thickness were prepared using a conventional microtome. The sections were stained with hematoxylin and eosin (H&E) and observed in a light field microscope. Histopathological criteria such as integrity of the nucleus, cytoplasm, cell membranes, as well as the degree of cellular damage and the interactions between trophozoites and intestinal or hepatic tissue were used, in order to evaluate the morphological characteristics of the tissue. The effect induced by treatment with the synthetic compounds and metronidazole on these morphological parameters was determined in both infected and control slices.

### Statistical analysis

The data from control and treatment groups were analyzed using t-test analysis. Values were considered significantly different if p < 0.05.The SPSS software version 21 was used.
